# Effects of Exercise on Depression and Anxiety in Lung Cancer Survivors: A Systematic Review and Meta-Analysis of Randomized Controlled Trials

**DOI:** 10.3390/curroncol32060304

**Published:** 2025-05-25

**Authors:** Cuiqing Zhao, Xifeng Tao, Bingkai Lei, Yifan Zhang, Gen Li, Yuanyuan Lv, Laikang Yu

**Affiliations:** 1 Division of Physical Education, Myongji University, Seoul 03674, Republic of Korea; cuiqingzhao521@163.com; 2 Beijing Key Laboratory of Sports Performance and Skill Assessment, Beijing Sport University, Beijing 100084, China; txf19983480529@126.com; 3 School of Physical Education, Xihua University, Chengdu 610039, China; leibingkai0520@163.com; 4 Department of Strength and Conditioning Assessment and Monitoring, Beijing Sport University, Beijing 100084, China; 18738314013@163.com; 5 School of Physical Education & Sports Science, South China Normal University, Guangzhou 510631, China; li287270242@163.com; 6 China Institute of Sport and Health Science, Beijing Sport University, Beijing 100084, China

**Keywords:** exercise, lung cancer, depression, anxiety

## Abstract

This study aims to investigate the effects of exercise on depression and anxiety in lung cancer survivors and identify the optimal exercise prescription for this population. Searches were conducted in PubMed, Web of Science, Cochrane Library, Embase, Scopus, CNKI, and Wanfang Data up to 7 January 2024. A meta-analysis was performed to calculate the standardized mean difference (SMD) and 95% confidence interval. Thirteen studies were included in this meta-analysis. Exercise significantly alleviated depression (SMD, −0.54; *p* = 0.002) and anxiety (SMD, −0.66; *p* = 0.0002) in lung cancer survivors. Subgroup analyses showed that aerobic exercise, exercise conducted >3 times per week, <60 min per session, and ≥180 min per week, were more effective in alleviating depression and anxiety, particularly in middle-aged individuals. In conclusion, exercise alleviates depression and anxiety in lung cancer survivors, particularly those who are middle-aged, and aerobic exercise may be the most effective intervention. This meta-analysis provides clinicians with evidence to recommend that lung cancer survivors engage in exercise more than three times per week, with each session lasting less than 60 min, aiming to achieve a total of 180 min per week by increasing the frequency of exercise.

## 1. Introduction

Lung cancer is the most prevalent form of cancer, significantly contributing to the escalating global public health burden [1,2,3]. In recent years, cancer-related deaths, totaling 2,397,700, have increased by 21.6%, with lung cancer ranking among the top five cancers with the highest mortality rates [3]. Lung cancer survivors commonly exhibit clinical symptoms such as hemoptysis, chest pain, fatigue, and weight loss [4,5]. Notably, these clinical symptoms often lead to emotional issues, including depression and anxiety [6], frequently adding an additional burden onto survivors during the treatment process. However, depression and anxiety are frequently overlooked in clinical assessments. Additionally, studies have shown that over 40% of all lung cancer survivors experience psychological distress, representing one of the highest prevalence rates among all cancers [7]. Emotional distress can reduce adherence to subsequent adjuvant therapies and impair survivors’ quality of life [8]. Moreover, it is negatively correlated with the survival rates of cancer survivors [9]. Therefore, prompt intervention, as recommended by the National Comprehensive Cancer Network [10], should be applied when necessary. A longitudinal study conducted in early adulthood indicated that high levels of anxiety may elevate the risk of cancer-related mortality [11]. Consequently, the negative impact of depression and anxiety must be thoroughly considered when devising treatment plans for lung cancer survivors.

Exercise, as a non-pharmacological intervention, has been proven to benefit individuals with depression and anxiety [12], as evidenced by recent studies [13,14]. For example, He et al. [14] showed positive effects of exercise interventions on depressive symptoms across various age groups. Recently, a network meta-analysis further demonstrated the equivalence of aerobic exercise, resistance exercise, and mind–body exercises in alleviating depressive symptoms in people over 65 years of age [15]. Additionally, a previous study demonstrated that 20 weeks of resistance exercise significantly reduced depressive symptoms in people with Parkinson’s disease [16]. Furthermore, evidence indicates that exercise can alleviate both depression and anxiety in people with breast cancer [17]. Moreover, one recent review concluded that aerobic exercise is the most effective mode for improving anxiety symptoms [18].

Despite these benefits of exercise, considerable debate persists regarding the effectiveness of exercise for depression and anxiety in individuals with lung cancer, including optimal exercise dosage. For example, an 8-week exercise intervention incorporating both Baduanjin and resistance exercise was shown to significantly alleviate depression and anxiety in lung cancer survivors [19]. Conversely, Temel et al. [20] demonstrated that a structured exercise regimen for people with advanced non-small cell lung cancer did not lead to improvements in depression or anxiety. In addition, it was found that 6 weeks of multidimensional exercise did not significantly improve depressive symptoms, although anxiety scores were reduced [21]. Furthermore, a meta-analysis indicated that exercise has the potential to improve depression and anxiety in individuals with advanced lung cancer [22]. However, that meta-analysis included only two studies related to depression and anxiety, and the assessment measures were not distinctly categorized. Consequently, the current study builds upon this foundation by further distinguishing between depression and anxiety.

Moreover, a recent meta-analysis conducted by Su et al. [23] demonstrated that exercise interventions may reduce depression and anxiety levels in lung cancer survivors. However, that study did not focus on specific exercise moderators, leaving it uncertain how these individuals should exercise. Therefore, the current study was conducted to investigate the effects of exercise on depression and anxiety in lung cancer survivors and identify the optimal exercise prescription for this population.

## 2. Materials and Methods

This meta-analysis adhered to the Preferred Reporting Items for Systematic Reviews and Meta-Analyses (PRISMA) guidelines, and the protocol was registered on PROSPERO with the registration number CRD42024500407.

### 2.1. Search Strategy

A comprehensive search was performed across 7 databases, including PubMed, Web of Science, Cochrane Library, Embase, Scopus, China National Knowledge Internet (CNKI), and Wanfang Data, up to 7 January 2024. The initial search included the following specific keywords and MESH terms: exercise, cancer, depression, and anxiety. Additionally, the reference lists of all identified reviews were hand-searched. Two authors (CZ and XT) independently completed the process using a standardized form. In cases of disagreement between the two authors, a third author (LY) joined the discussion until a consensus was reached.

### 2.2. Eligibility Criteria

The following criteria were considered: (1) randomized controlled trial (RCT) design; (2) lung cancer survivors as subjects; (3) the presence of both an intervention group and a control group; and (4) inclusion of a metric for depression or anxiety outcomes.

The exclusion criteria included (1) duplicate publications; (2) conference papers; (3) incomplete data reporting; (4) acute exercise interventions; and (5) animal studies.

### 2.3. Data Extraction

Two authors (C.Z. and X.T.) independently extracted data, focusing on: (1) study details, such as the first author’s last name and the year of publication; (2) specifics of the intervention, including type of intervention, frequency, intervention duration, and session duration; (3) participant information, such as sample size, gender, and age; and (4) treatment outcomes. In cases where the results were incomplete, the corresponding authors were contacted via email for further information.

### 2.4. Methodological Quality Assessment

The methodological quality of the included studies was assessed using the Cochrane risk of bias criteria [24], mainly from 7 aspects: random sequence generation (selection bias), allocation concealment (selection bias), blinding of participants, personnel (performance bias), outcome-blind assessment (detection bias), incomplete result data (loss bias), selective reporting (report bias), and other biases. The assessment of methodological quality was independently conducted by two authors (C.Z. and X.T.), with any disagreements being discussed and resolved through consensus with a third author (L.Y.).

### 2.5. Statistical Analysis

A meta-analysis was performed using Review Manager software (version 5.4). Changes in mean and standard deviation (SD) were calculated for depression and anxiety. For studies reporting the standard error (SE) or 95% confidence interval (CI), SD was derived through conversion [25,26]. Data were pooled using either fixed-effects or random-effects models to determine the standardized mean difference (SMD) and 95% CI. Heterogeneity was assessed using the I^2^ statistic [27], with values of 0%, 25%, 50%, and 75% interpreted as indicating no, low, moderate, and high heterogeneity. Where heterogeneity was high (I^2^ > 60%), subgroup analysis and sensitivity analysis were performed to explain the results [28]. Funnel plots were used to scrutinize potential publication bias. The significance level was set at *p* < 0.05.

## 3. Results

### 3.1. Study Selection

**Figure 1:**
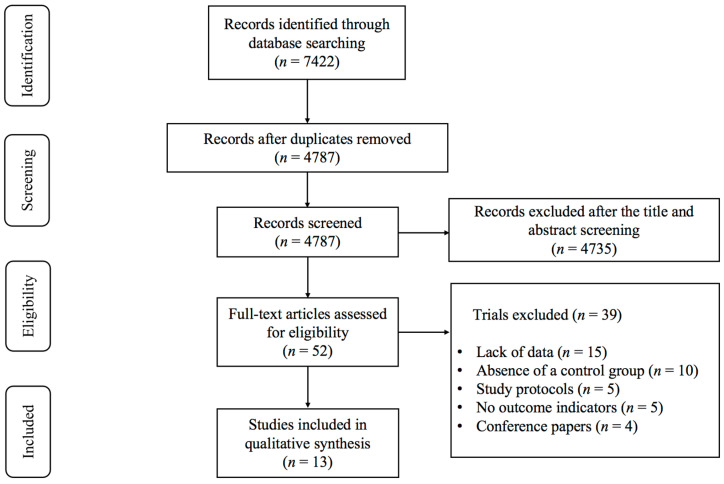
PRISMA flowchart of study selection.

As illustrated in Figure 1, 7422 records were identified, consisting of 7270 articles from English databases and 152 from Chinese databases. After removing duplicates, 4787 studies remained. Following the screening of titles and abstracts, 4735 studies were deemed ineligible for inclusion, leaving 52 articles selected for full-text assessment. A total of 39 studies were excluded after a full-text review of these 52 studies, for the following reasons: (1) lack of data (*n* = 15); (2) absence of a control group (*n* = 10); (3) study protocols (*n* = 5); (4) no outcome indicators (*n* = 5); and (5) conference papers (*n* = 4). Ultimately, 13 studies [19,29,30,31,32,33,34,35,36,37,38,39,40] investigating the effects of exercise on depression and anxiety in lung cancer survivors were included in the meta-analysis.

### 3.2. Characteristics of the Included Studies

The primary characteristics of the interventions and participants are presented in Table S1. A total of 449 participants across 15 exercise intervention groups and 441 participants across 13 control groups were included in the studies. The participants’ mean age ranged from 48.1 to 68.0 years. Participants in seven studies [19,33,34,35,36,37,39] were included, with a mean age of 45–60 years, while participants in another six studies [29,30,31,32,38,40] were included, with a mean age of ≥60 years. Interventions specified aerobic exercise [29,30,33,34,35,37,39,40], combined exercise [19,31,38,40], and high-intensity interval training (HIIT) [36]. The total duration of intervention ranged from 4 to 12 weeks, the frequency of intervention per week was 2 to 14 times, and the minutes of intervention per session ranged from 15 to 90 min. In terms of results, 12 studies addressed depression, while 12 studies examined anxiety.

### 3.3. Meta-Analysis Results

**Figure 2:**
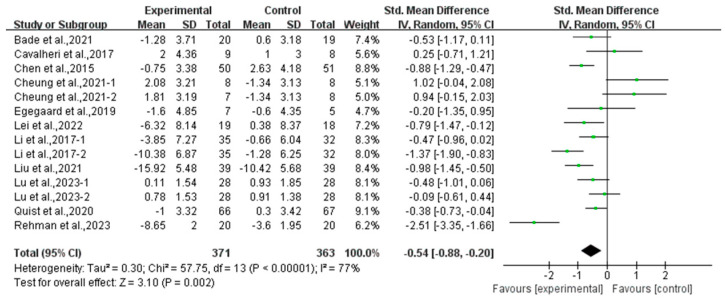
Meta-analysis results of the effects of exercise on depression in lung cancer survivors.

**Figure 3:**
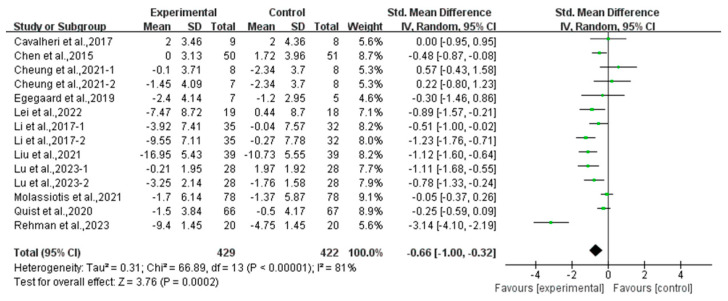
Meta-analysis results of the effects of exercise on anxiety in lung cancer survivors.

Compared with the control group, exercise had a positive effect on alleviating depression (SMD, −0.54; 95% CI, −0.88 to −0.20, *p* = 0.002, I^2^ = 77%, Figure 2) and anxiety (SMD, −0.66; 95% CI, −1.00 to −0.32, *p* = 0.0002, I^2^ = 81%, Figure 3) in lung cancer survivors.

### 3.4. Subgroup Analysis

#### 3.4.1. Effects of Various Exercise Moderators on Depression in Lung Cancer Survivors

**Figure 4:**
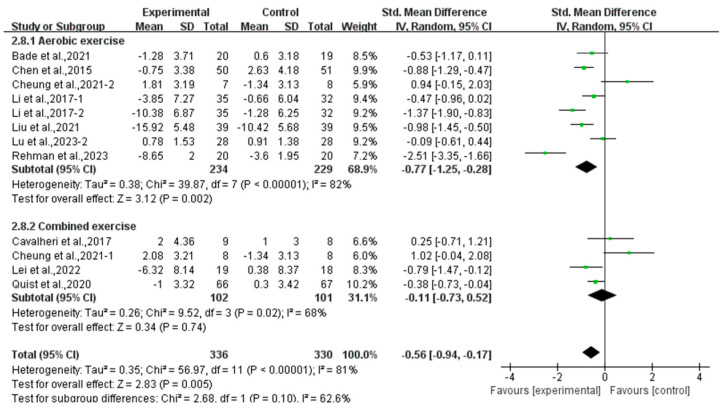
Meta-analysis results of the effects of types of intervention on depression in lung cancer survivors.

Subgroup analysis showed that aerobic exercise (SMD, −0.77; 95% CI, −1.25 to −0.28, *p* = 0.002, I^2^ = 82%) significantly alleviated depression, while combined exercise had no significant effect on depression (SMD, −0.11; 95% CI, −0.73 to 0.52, *p* = 0.74, I^2^ = 68%, Figure 4) in lung cancer survivors.

**Figure 5:**
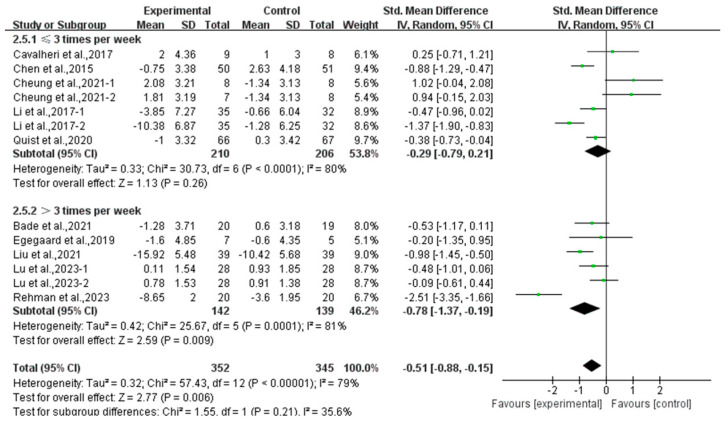
Meta-analysis results of the effects of frequency of exercise on depression in lung cancer survivors.

In addition, a subgroup analysis based on intervention frequency revealed that exercise conducted >3 times per week significantly alleviated depression (SMD, −0.78; 95% CI, −1.37 to −0.19, *p* = 0.009, I^2^ = 81%), while exercise conducted for ≤3 times per week had no significant effect on depression (SMD, −0.29; 95% CI, −0.79 to 0.21, *p* = 0.26, I^2^ = 80%, Figure 5) in lung cancer survivors.

**Figure 6:**
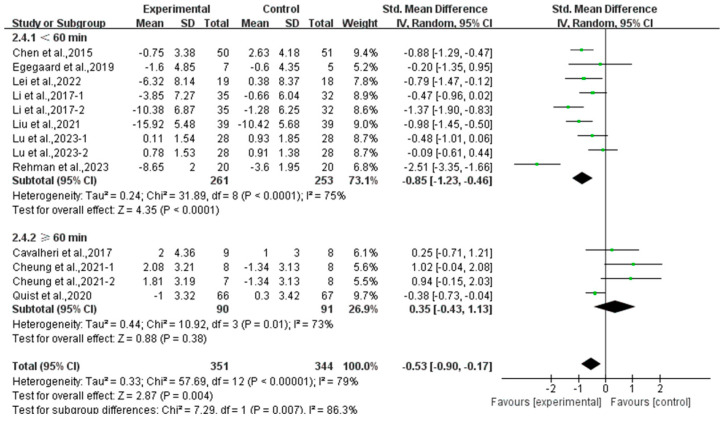
Meta-analysis results of the effects of duration of exercise per session on depression in lung cancer survivors.

Furthermore, analyzing the subgroup by session duration, exercise lasting <60 min per session significantly alleviated depression (SMD, −0.85; 95% CI, −1.23 to −0.46, *p* < 0.0001, I^2^ = 75%), while exercise lasting ≥60 min per session had no significant effect on depression (SMD, 0.35; 95% CI, −0.43 to 1.13, *p* = 0.38, I^2^ = 73%, Figure 6) in lung cancer survivors.

**Figure 7:**
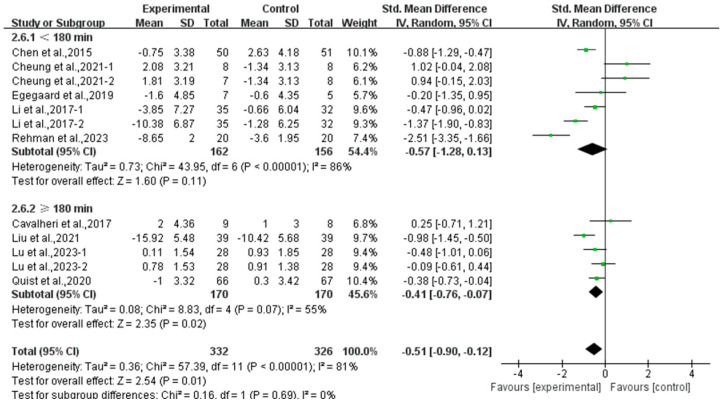
Meta-analysis results of the effects of duration of exercise per week on depression in lung cancer survivors.

Moreover, a subgroup analysis according to weekly time found that exercise conducted for ≥180 min per week significantly alleviated depression (SMD, −0.41; 95% CI, −0.76 to −0.07, *p* = 0.02, I^2^ = 55%), while exercise conducted for <180 min per week had no significant effect on depression (SMD, −0.57; 95% CI, −1.28 to 0.13, *p* = 0.11, I^2^ = 86%, Figure 7) in lung cancer survivors.

**Figure 8:**
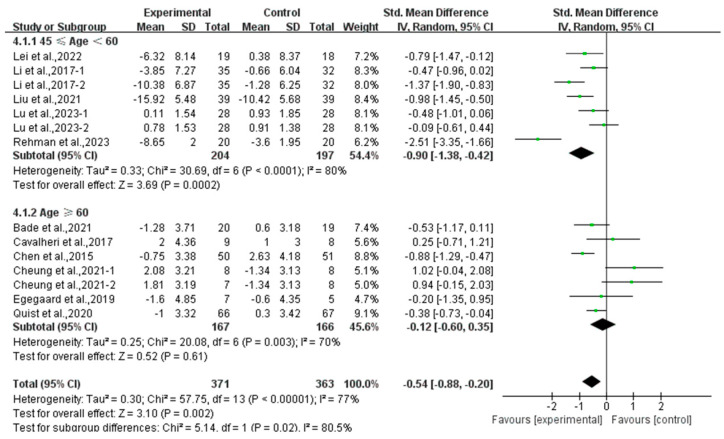
Meta-analysis results of the effects of exercise on depression in middle-aged and elderly lung cancer survivors.

Finally, exercise significantly alleviated depression in middle-aged lung cancer survivors (SMD, −0.90; 95% CI, −1.38 to −0.42, *p* = 0.0002, I^2^ = 80%), while exercise had no significant effect on depression in elderly lung cancer survivors (SMD, −0.12; 95% CI, −0.60 to 0.35, *p* = 0.61, I^2^ = 70%, Figure 8).

#### 3.4.2. Effects of Various Exercise Moderators on Anxiety in Lung Cancer Survivors

**Figure 9:**
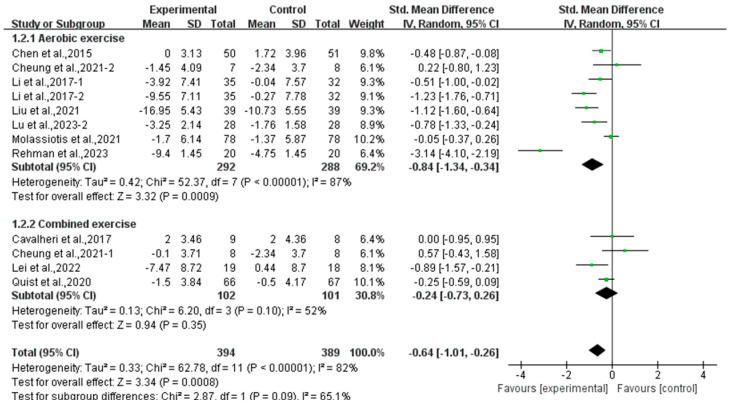
Meta-analysis results of the effects of types of intervention on anxiety in lung cancer survivors.

Subgroup analysis showed that aerobic exercise significantly alleviated anxiety (SMD, −0.84; 95% CI, −1.34 to −0.34, *p* = 0.0009, I^2^ = 87%), while combined exercise had no significant effect on anxiety (SMD, −0.24; 95% CI, −0.73 to 0.26, *p* = 0.35, I^2^ = 52%, Figure 9) in lung cancer survivors.

**Figure 10:**
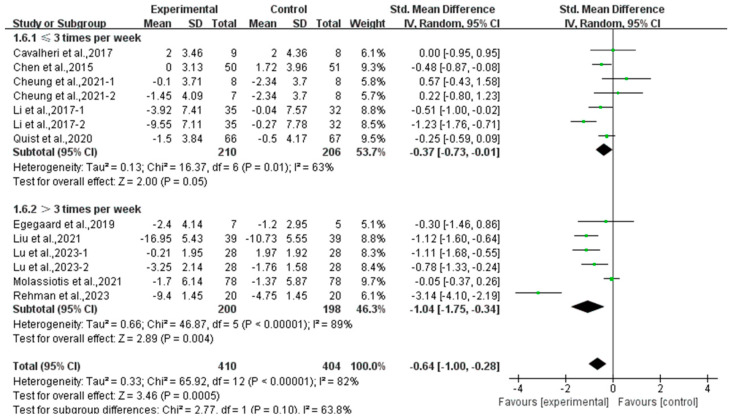
Meta-analysis results of the effects of frequency of exercise on anxiety in lung cancer survivors.

In addition, a subgroup analysis based on intervention frequency revealed that exercise conducted ≤3 times per week (SMD, −0.37; 95% CI, −0.73 to −0.01, *p* = 0.05, I^2^ = 63%) and >3 times per week (SMD, −1.04; 95% CI, −1.75 to −0.34, *p* = 0.004, I^2^ = 89%, Figure 10) significantly alleviated anxiety in lung cancer survivors, with exercise conducted >3 times per week showing a better effect.

**Figure 11:**
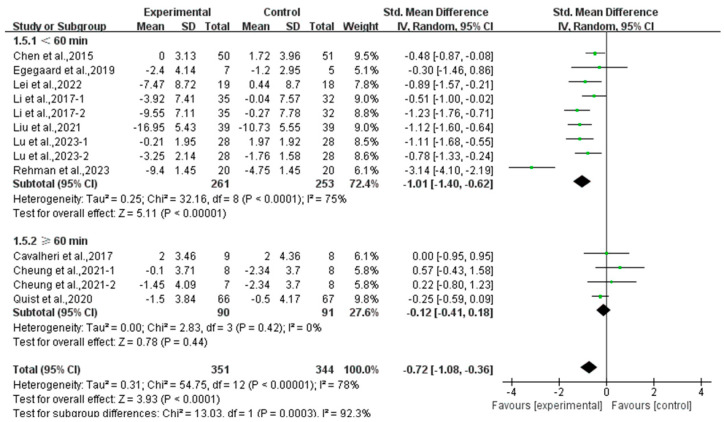
Meta-analysis results of the effects of duration of exercise per session on anxiety in lung cancer survivors.

Furthermore, analyzing the subgroup by session duration, exercise lasting <60 min per session significantly alleviated anxiety (SMD, −1.01; 95% CI, −1.40 to −0.62, *p* < 0.00001, I^2^ = 75%), while exercise lasting ≥60 min per session had no significant effect on anxiety (SMD, −0.12; 95% CI, −0.41 to 0.18, *p* = 0.44, I^2^ = 0%, Figure 11) in lung cancer survivors.

**Figure 12:**
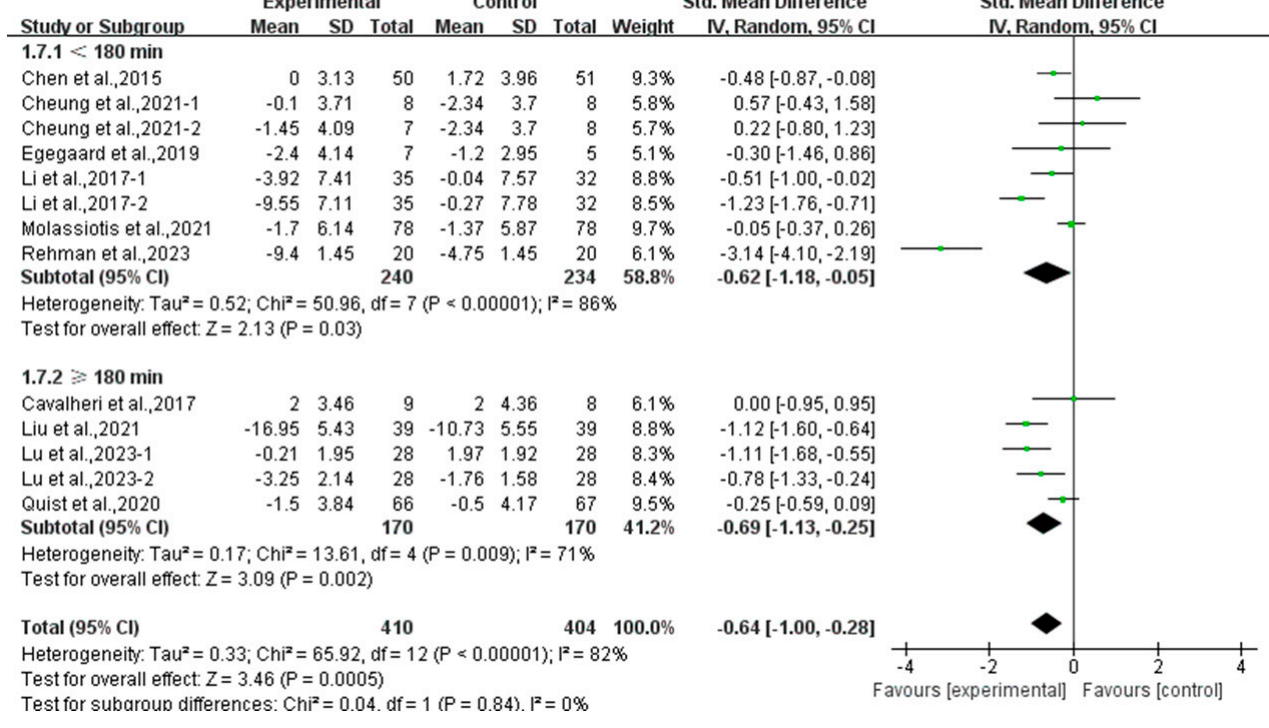
Meta-analysis results of the effects of duration of exercise per week on anxiety in lung cancer survivors.

Moreover, a subgroup analysis according to weekly time found that exercise conducted for <180 min per week (SMD, −0.62; 95% CI, −1.18 to −0.05, *p* = 0.03, I^2^ = 86%) and ≥180 min per week significantly alleviated anxiety (SMD, −0.69; 95% CI, −1.13 to −0.25, *p* = 0.002, I^2^ = 71%, Figure 12) in lung cancer survivors, with exercise conducted for ≥180 min per week showing a better effect.

**Figure 13:**
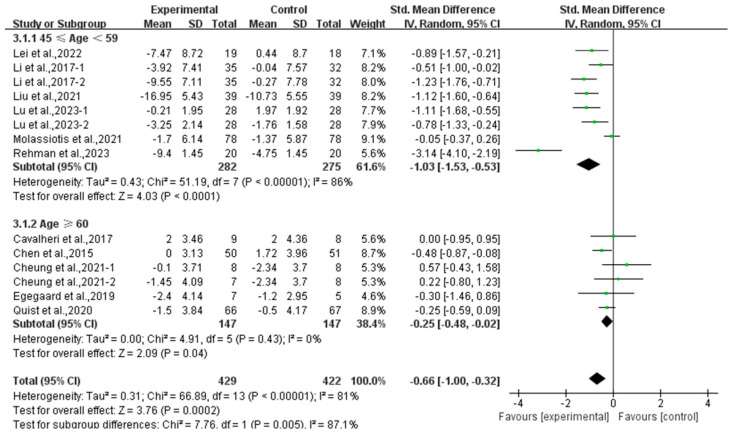
Meta-analysis results of the effects of exercise on anxiety in middle-aged and elderly lung cancer survivors.

Finally, exercise significantly alleviated anxiety in middle-aged (SMD, −1.03; 95% CI, −1.53 to −0.53, *p* < 0.0001, I^2^ = 86%) and elderly lung cancer survivors (SMD, −0.25; 95% CI, −0.48 to −0.02, *p* = 0.04, I^2^ = 0%, Figure 13), with a more pronounced effect observed in middle-aged lung cancer survivors.

### 3.5. Risk of Bias

This study employed the Cochrane risk of bias criteria for quality assessment, which is based on six factors: selection bias, performance bias, detection bias, attrition bias, reporting bias, and other biases. The quality of the included studies was classified into three levels based on the criteria: low risk, high risk, and unclear (Figure S1).

### 3.6. Publication Bias

Funnel plots were visually inspected to assess publication bias, revealing asymmetry for both depression (Figure S2) and anxiety (Figure S3). Egger’s test indicated that small sample size did not significantly influence the combined effect sizes for depression (*p* = 0.439) or anxiety (*p* = 0.336, Table S2).

### 3.7. Sensitivity Analysis

Sensitivity analysis showed that the positive effects of exercise on depression (Figure S4) and anxiety (Figure S5) in lung cancer survivors remained stable in both direction and magnitude, regardless of the exclusion of any individual studies.

## 4. Discussion

### 4.1. Main Findings

To our knowledge, this is the first systematic review and meta-analysis to explore the effects of exercise on depression and anxiety in lung cancer survivors. Thirteen studies were included and the findings indicated that exercise significantly alleviated depression and anxiety in lung cancer survivors. Subgroup analyses showed that aerobic exercise, exercise conducted >3 times per week, <60 min per session, and ≥180 min per week were more effective in alleviating depression and anxiety in lung cancer survivors, particularly in middle-aged individuals.

### 4.2. Effects of Exercise on Depression and Anxiety in Lung Cancer Survivors

It is widely recognized that exercise can serve as an effective complement or alternative to both pharmacological and psychological therapies [14,41,42,43]. As a non-invasive and non-pharmacological intervention, exercise has been shown to effectively alleviate depressive symptoms, as evidenced by previous studies [44,45]. A meta-analysis by Singh et al. [46] showed that physical activity is particularly effective in alleviating symptoms of depression and anxiety in adults. In addition, a systematic review also indicated that exercise can alleviate depression in lung cancer survivors [47]. Furthermore, in an observational study of 8098 adults, individuals who exercised regularly exhibited a lower risk of being diagnosed with an anxiety disorder compared to those who were sedentary [48].

Both qualitative and quantitative studies have illustrated the benefits of exercise on depression and anxiety. Although clear causal mechanisms were not uncovered, the trends observed in the data are valuable for generating hypotheses. The beneficial effects of exercise on depression and anxiety are likely to be attributable to a combination of various neurophysiological and psychological mechanisms. Current research has identified several potential physiological mechanisms, including the regulation of hypothalamic–pituitary–adrenal (HPA) axis activity, enhanced monoamine system function, promotion of new neuronal growth in the hippocampus, reduction of systemic inflammation, and increased levels of opioids and brain-derived neurotrophic factor (BDNF) [49,50,51].

Dysfunction within the HPA axis has been linked to a range of symptoms, including disorders of the sympathetic nervous system, hyperarousal, and mental illness such as depression and anxiety [52,53]. Animal studies found that 6 weeks of voluntary wheel running regulated HPA axis activity [54,55]. Additionally, exercise can increase levels of β-endorphins [56,57], vascular endothelial growth factor (VEGF) [58,59], BDNF [60], and serotonin (5-HT) [61]. β-endorphins facilitate the generation of new neurons in the dentate gyrus [62], while 5-HT has been shown to enhance cellular proliferation and neurogenesis in this region among adults [63]. VEGF [58] and BDNF [64] are acknowledged for their roles in supporting neuronal survival. Therefore, the therapeutic effects of exercise on depression and anxiety may be linked to its potential to enhance neurogenesis in the adult hippocampus through these mechanisms [51].

Furthermore, the pathology of depression and anxiety is associated with abnormalities in the function of monoamines in the brain [49]. Numerous studies have found that exercise can increase the availability of central monoamines, such as dopamine [65], norepinephrine [66], and 5-HT [67], thereby regulating depression and anxiety. Concurrently, it helps lower the levels of inflammatory factors like interleukin-17 (IL-17) and interleukin-1 beta (IL-1β) [68]. Moreover, exercise plays a vital role in increasing endogenous opioid activity in the central and peripheral nervous system [56], thereby further supporting mental health. In addition to physiological mechanisms, psychosocial factors, such as self-efficacy, are recognized as playing a crucial role [49]. Numerous studies have suggested that the enhancement of self-efficacy through exercise may alleviate negative emotions such as depression and anxiety [69].

Conversely, Cavalheri et al. [31] discovered that anxiety and depression did not demonstrate improvements following exercise. This may be attributed to a sample size of fewer than 30 participants and the relatively low baseline scores for depression and anxiety. A recent review by Bartley et al. [70] indicated that aerobic exercise is ineffective in alleviating anxiety, which contradicts the findings of the current study. However, it is worth noting that the population included in Bartley et al.’s study was not specifically subdivided, unlike the population of lung cancer survivors in this study. There is considerable debate in the existing research, which may be attributed to discrepancies in the characteristics of the research subjects, as well as the physical condition, rehabilitation capacity, and recovery potential of patients across different age groups. Moreover, the type of exercise and the duration of the intervention may result in disparate outcomes with respect to depression.

### 4.3. Effects of Various Exercise Moderators on Depression and Anxiety in Lung Cancer Survivors

In order to determine the optimal exercise prescription for lung cancer survivors, we conducted subgroup analyses based on the type of intervention, session duration, frequency, weekly time, and participants’ age.

Through subgroup analysis of type of intervention, we found that only aerobic exercise exhibited positive effects in alleviating depression and anxiety in lung cancer survivors, aligning with the results of a previous study [71] showing that there was a negative correlation between aerobic exercise and depression and anxiety in cancer survivors. Additionally, a meta-analysis indicated that Tai Chi and Qigong, as forms of aerobic exercise, can help regulate depression levels in cancer survivors [72]. Meanwhile, a recent review has demonstrated that aerobic exercise has a significant effect on alleviating anxiety symptoms [73].

Numerous observational studies have shown a negative correlation between exercise, especially aerobic exercise, and anxiety symptoms [18,74,75]. Its effectiveness in alleviating depression and anxiety may be attributed to its ease of implementation and widespread feasibility. Cancer survivors typically exhibit significantly lower levels of physical functioning compared with the general population [76]. Aerobic exercise is characterized by lower intensity compared with combined exercise. Furthermore, aerobic exercise may be more tolerable for cancer survivors, and it has been established that aerobic exercise is both safe and effective [77]. Notably, a meta-analysis examining the effects of HIIT on depression and anxiety reported no significant improvements [78]. Paolucci et al. [79] found that HIIT resulted in elevated levels of pro-inflammatory cytokines, including tumor necrosis factor alpha (TNF-α) and interleukin-6 (IL-6), along with increased perceived stress. In contrast, moderate-intensity continuous aerobic exercise did not induce such effects. Resistance exercise is often associated with mild muscle pain and falls experienced by participants [15], which may contribute to the lack of significant effects from combined exercise.

It is noteworthy that exercise conducted more than three times per week significantly alleviated depression and anxiety in lung cancer survivors, which is consistent with previous studies [80,81], and the intervention effect was enhanced with a higher exercise frequency. This phenomenon may be attributed to the moderating effect of exercise frequency on endogenous opioids, with studies showing that regular exercise four times a week enhances endorphin release [82]. Other studies have shown that exercising more than three times a week can significantly improve depression and anxiety in lung cancer survivors [39]. Additionally, Temel et al. [20] suggested that exercising twice a week does not significantly improve depression and anxiety. Thus, exercise more than three times per week may produce more favorable outcomes in terms of depression and anxiety.

In addition, a subgroup analysis of session duration revealed that exercise lasting <60 min per session was more effective in alleviating depression and anxiety in lung cancer survivors, which is in agreement with the results of Rethorst et al. [83], showing that durations of 45 to 59 min produced greater antidepressant benefits than longer bouts of activity. A previous study showed that extended exercise sessions exceeding 60 min may lead to considerable muscle fatigue and increased concentrations of lactic acid [84]. It has been demonstrated that lung cancer survivors typically exhibit lower exercise tolerance [85], which may hinder their ability to engage in prolonged exercise during each session.

Considering only the frequency and session duration is insufficient to account for the influence of other variables. The World Health Organization (WHO) recommends that individuals engage in 150–300 min of moderate-intensity aerobic exercise per week [86]. Therefore, we combined frequency and session duration to calculate the weekly time. Our subgroup analysis showed that exercise lasting ≥180 min per week was more effective in alleviating depression and anxiety in lung cancer survivors. This finding is consistent with previous studies [87,88,89], suggesting that increasing the frequency of interventions may necessitate a reduction in the duration of individual sessions while ensuring that the total exercise volume is remains at a minimum of 180 min per week. This dose–response relationship may be more effective in alleviating depression and anxiety in lung cancer survivors, ensuring both safety and improved outcomes.

Finally, this study indicated that exercise had a more pronounced effect on alleviating depression and anxiety in middle-aged lung cancer survivors. However, as individuals age, several factors come into play that can influence the efficacy of exercise interventions. Firstly, lower tolerance for exercise becomes more prevalent among older adults, which may limit their ability to engage in intense or prolonged physical activities [90]. This decline in exercise tolerance can be attributed to age-related changes in muscle mass, cardiovascular efficiency, and overall physical strength. Secondly, increased susceptibility to fatigue is another challenge faced by older lung cancer survivors. Fatigue is a common side effect of both the disease itself and its treatment, and it can exacerbate the physical and mental strain experienced by these individuals [91]. Lastly, decreased adherence to exercise among older adults can also undermine the benefits of exercise interventions. This may be due to various factors, including lack of social support, mobility limitations, or simply a shift in priorities as individuals prioritize other aspects of their health and daily living [92].

### 4.4. Limitations of This Study

This study has the following limitations. Firstly, the results of this study should be interpreted with caution due to the potential risk of bias. The trials evaluated were at a high risk of performance bias because blinding participants in exercise interventions is not feasible unless a more rigorously controlled comparison design is employed to assess the effects of the intervention. Secondly, some of the studies that were included did not report the intensity of exercise interventions, thus limiting the understanding of how exercise intensity influences depression and anxiety in lung cancer survivors. Lastly, given the limited number of studies on exercise interventions and the absence of resistance exercise among the exercise modalities, further research is necessary to address these gaps.

## 5. Conclusions

Exercise alleviated depression and anxiety in lung cancer survivors, particularly those who are middle-aged, and aerobic exercise may be the most effective intervention. To alleviate depression and anxiety, this meta-analysis provides clinicians with evidence to recommend that lung cancer survivors engage in exercise more than three times per week, with each session lasting less than 60 min, aiming to achieve a total of 180 min per week by increasing the frequency of exercise.

## Data Availability

All data generated or analyzed during this study are included in the article/Supplementary Materials.

## Supplementary Materials

The following supporting information can be downloaded at https://www.mdpi.com/article/10.3390/curroncol32060304/s1, Figure S1: Results of Cochrane risk of bias tool; Figure S2: Funnel plot (depression); Figure S3: Funnel plot (anxiety); Figure S4: Sensitivity analyses results (depression); Figure S5: Sensitivity analyses results (anxiety); Table S1: Characteristics of studies included in this meta-analysis; Table S2: Results of Egger’s test.

## Abbreviations

The following abbreviations are used in this manuscript:

PRISMA	Preferred Reporting Items for Systematic Evaluation and Meta-Analyses
CNKI	China National Knowledge Internet
RCT	Randomized controlled trial
SD	Standard deviation
SE	Standard error
CI	Confidence interval
SMD	Standardized mean difference
HIIT	High-intensity interval training
HPA	Hypothalamic–pituitary–adrenal
BDNF	Brain-derived neurotrophic factor
VEGF	Vascular endothelial growth factor
5-HT	Serotonin
IL-17	Interleukin-17
IL-1β	Interleukin-1 beta
TNF-α	Tumor necrosis factor alpha
IL-6	Interleukin-6
WHO	World Health Organization
