# Supplementary material for: Effects of Exercise on Depression and Anxiety in Lung Cancer Survivors: A Systematic Review and Meta-Analysis of Randomized Controlled Trials
Source: Curr Oncol. 2025 May 25;32(6):304. doi: 10.3390/curroncol32060304 (PMC12191834; doi:10.3390/curroncol32060304)

Supplemental material

Effects of Exercise on Depression and Anxiety in Lung Cancer Survivors: A Systematic Review and Meta-analysis of Randomized Controlled Trials

Table S1. Characteristics of studies included in this meta-analysis..........2

Table S2. Results of Egger's test...4

Figure S1. Results of Cochrane risk of bias tool.....................................5

Figure S2. Funnel plot (depression)...6

Figure S3. Funnel plot (anxiety)...7

Figure S4. Sensitivity analyses results (depression).................................8

Figure S5. Sensitivity analyses results (anxiety)......................................9

Table S1. Characteristics of studies included in this meta-analysis

Study	Participant			Exercise intervention				Results	
	Gender (M/F)	Age (y)	Lung cancer type	Type	Duration	Frequency	Session duration	Outcomes	Measures
Bade et al. (2021)	IG: 20/0<br>CG: 19/0	IG: 66.55 ± 7.28<br>CG: 63.20 ± 9.80	NSLCC	Physical activity	12 weeks	7	Not clear	Depression	PHQ-9
Cavalheri et al. (2017)	IG: 3/6<br>CG: 2/6	IG: 66 ± 10<br>CG: 68 ± 9	NSCLC	Aerobic training, and stretching	8 weeks	3	60 min	Anxiety<br>Depression	HADS-A<br>HADS-D
Chen et al. (2015)	IG: 26/32<br>CG: 28/30	IG: 64.76 ± 11.28<br>CG: 63.57 ± 10.54	Not clear	Home-based, moderate-intensity walking-exercise	12 weeks	3	40 min	Anxiety<br>Depression	HADS-A<br>HADS-D
Cheung et al. (2021)	IG: 1/8<br>IG: 2/7<br>CG: 8/0	IG1: 61.00 ± 12.12<br>IG2: 61.11 ± 7.01<br>CG: 58.36 ± 9.32	Not clear	IG1: Aerobic exercise and strengthening exercises<br>IG2: Tai-chi	12 weeks	2	60 min	Anxiety<br>Depression	HADS-A<br>HADS-D
Egegaard et al. (2019)	IG: 7/0<br>CG: 5/0	IG: 64 ± 5.8<br>CG: 65 ± 4.7	NSCLC	Cycle ergometer interval training	7 weeks	5	20 min	Anxiety<br>Depression	HADS-A<br>HADS-D
Lei et al. (2022)	IG: 19/0<br>CG: 18/0	IG: 56.04 ± 11.67<br>CG: 58.03 ± 7.71	NSCLC	Baduanjin and resistance training	8 weeks	3-5	46-50 min	Anxiety<br>Depression	SAS<br>SDS
Li et al. (2017)	IG: 21/14<br>CG: 17/15	IG: 56.26 ± 7.86<br>CG: 56.09 ± 6.00	NSCLC	Baduanjin	6 weeks/12 weeks	3	20-30 min	Anxiety<br>Depression	SAS<br>SDS
Liu et al. (2021)	IG: 24/15<br>CG: 25/14	IG: 54.75 ± 7.32<br>CG: 54.89 ± 7.11	Not clear	Baduanjin	12 weeks	14	30 min	Anxiety<br>Depression	SAS<br>SDS
Lu et al. (2023)-1	IG: 12/16	IG: 56.32 ± 5.48	NSCLC	HIIT	⩽ 2 weeks	7	35 min	Anxiety	HADS-A

	CG: 13/15	CG: 58.04 ± 8.27						Depression	HADS-D
Lu et al. (2023)-2	IG: 28/0<br>CG: 28/0	IG: 56.67 ± 8.21<br>CG: 58.38 ± 7.40	NSCLC	Yoga	9-14 days	14	20 min	Anxiety<br>Depression	HADS-A<br>HADS-D
Molassiotis et al. (2021)	IG: 59/19<br>CG: 57/21	IG: 57.62 ± 9.63<br>CG: 56.06 ± 9.25	NSLCC<br>SLCC	Qigong	6 weeks	5	30-90 min	Anxiety	DASS21-A
Quist et al.（2020）	IG: 66/0<br>CG: 67/0	IG: 65.2 ± 8.2<br>CG: 63.5 ± 8.7	NSCLC<br>SCLC	Aerobic training, and stretching	12 weeks	2	90 min	Anxiety<br>Depression	HADS-A<br>HADS-D
Rehman et al. (2023)	IG: 11/9<br>CG: 13/7	IG: 48.1 ± 4.0<br>CG: 48.3 ± 3.8	NSCLC	Aerobic training	4 weeks	5	30 min	Anxiety<br>Depression	HADS-A<br>HADS-D

Abbreviation: M, male; F, female; y, year; IG, intervention groups; CG, control groups; NSCLC, non-small cell lung cancer; SLCC, small cell lung cancer; HIIT, high intensity interval training; PHQ-9, patient health questionnaire-9 depression scale; HADS-A, hospital anxiety and depression scale- anxiety; HADS-D, hospital anxiety and depression scale-depression; SAS, self-rating anxiety scale; SDS, self-rating depression scale; DASS21-A, depression anxiety stress scales 21-anxiety subscale.

Table S2. Results of Egger's test

Outcomes	Stad_EFF	Coef.	Stad. Err.	t	p > | t |	95% CI
**Depression**	Slope	-0.9877014	0.4984433	-1.98	0.071	-2.073716, 0.0983133
	Bias	1.378534	1.72251	0.80	0.439	-2.374493, 5.131562
**Anxiety**	Slope	-0.1555473	0. 4372557	-0.36	0.728	-1.108246, 0.797151
	Bias	-1.636761	1.634001	-1.00	0.336	-5.196945, 1.923422

Abbreviations: Coef, Coefficient; Std. Err, standard error; t, t-test statistic; p, probability; CI, confidence interval.

Figure S1. Results of Cochrane risk of bias tool

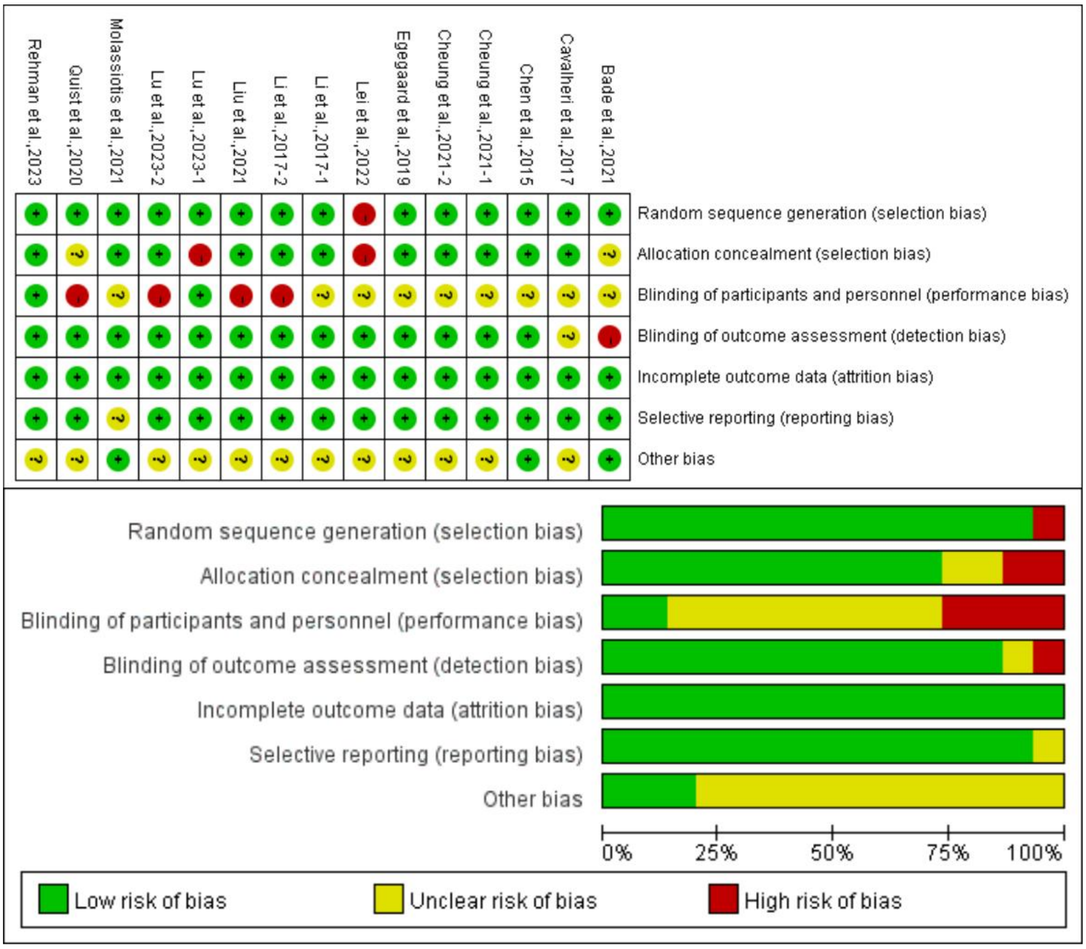

Figure S2. Funnel plot (depression)

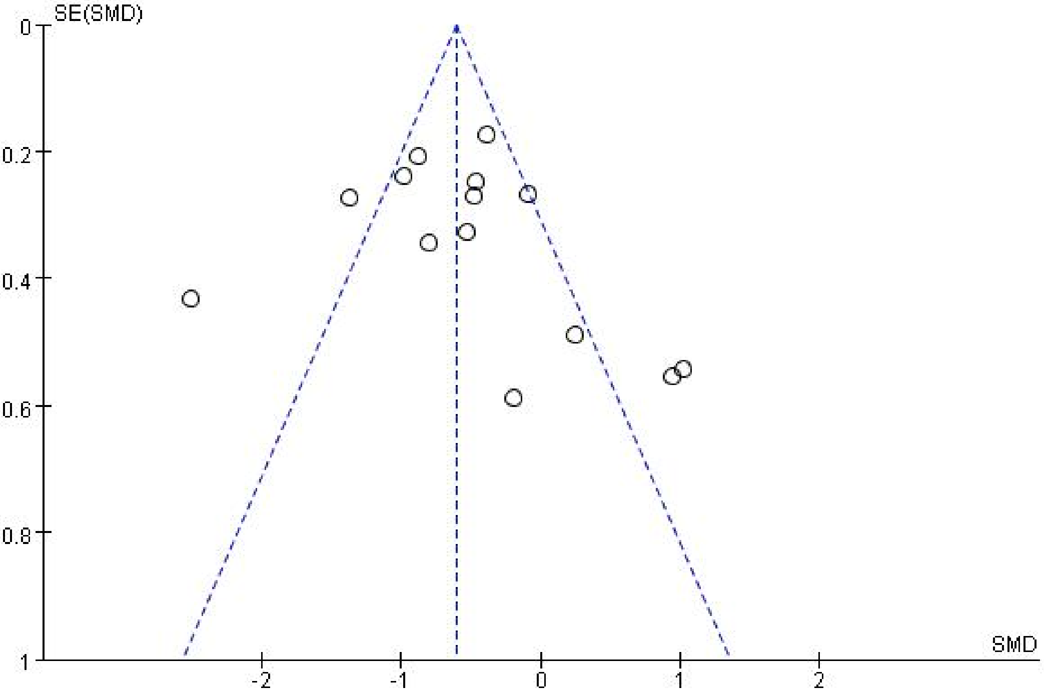

Figure S3. Funnel plot (anxiety)

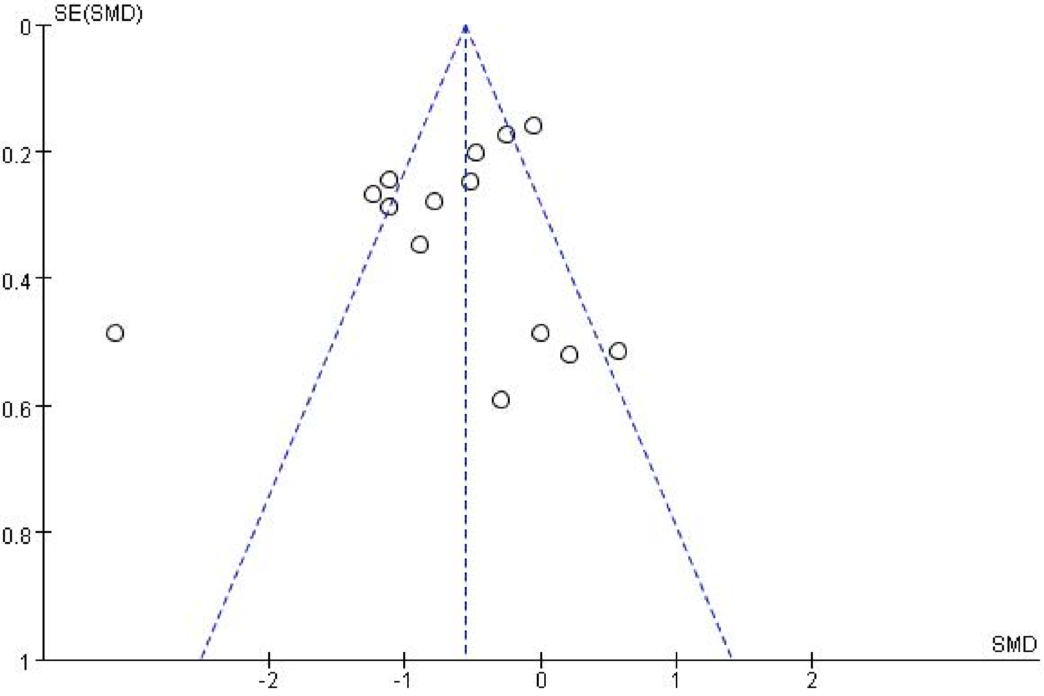

Figure S4. Sensitivity analyses results (depression)

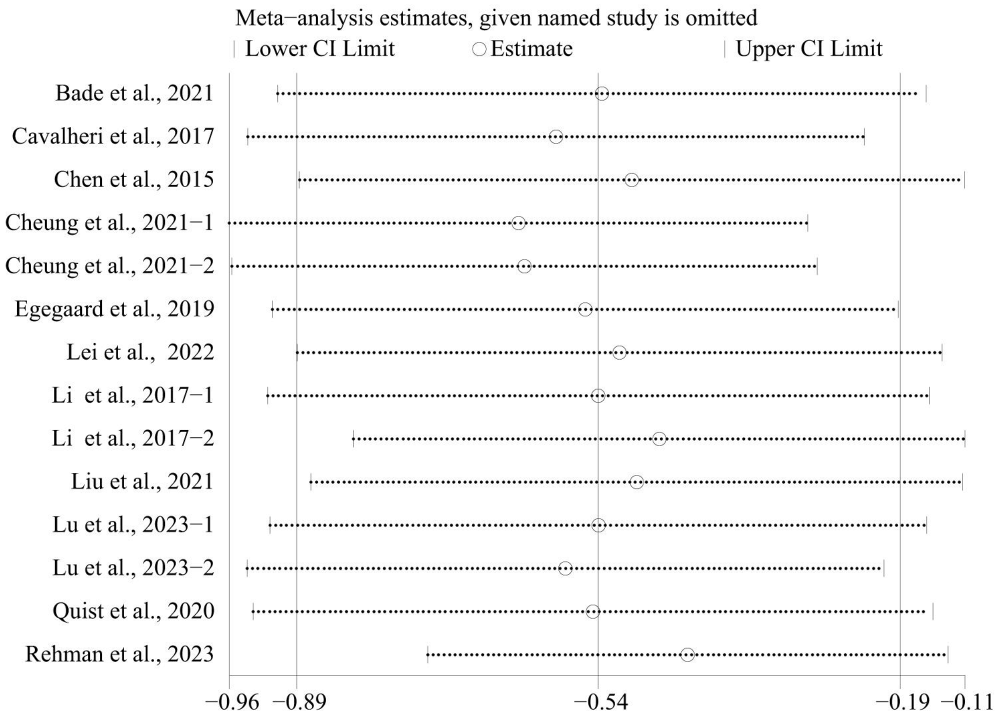

Figure S5. Sensitivity analyses results (anxiety)

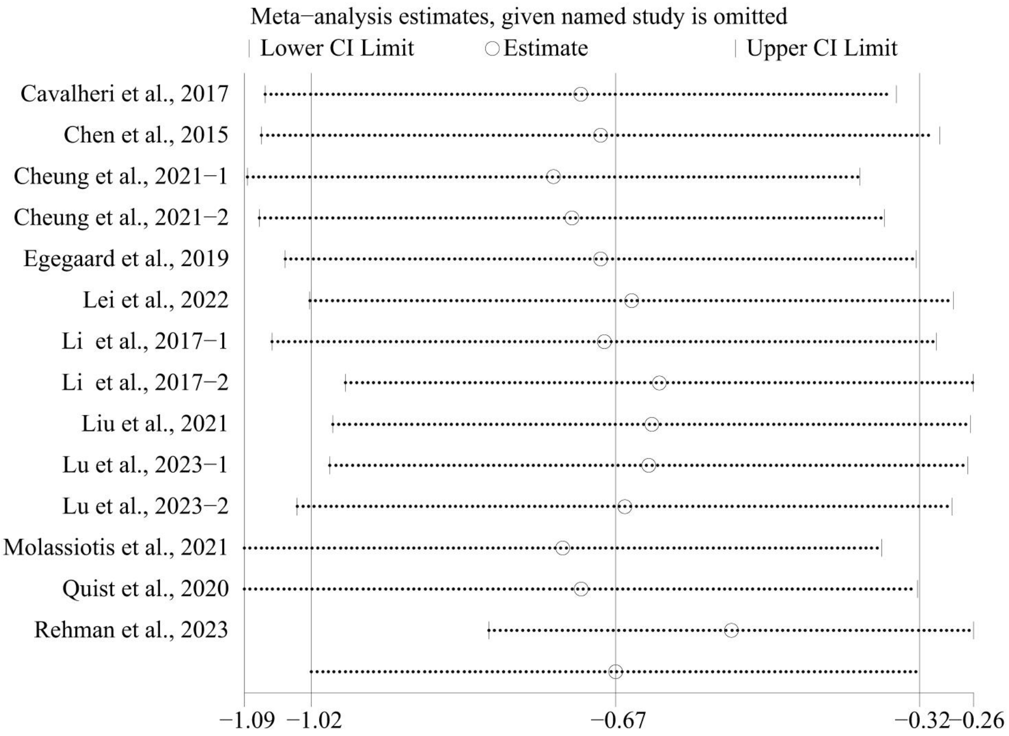

Supplement: Supplementary file 1 [file curroncol-32-00304-s001.zip › curroncol-3588469-supplementary.pdf]
